# Influence of L-Carnitine on Stored Rat Blood: A Study on Plasma

**DOI:** 10.4274/tjh.2016.0343

**Published:** 2017-12-01

**Authors:** Carl Hsieh, Vani Rajashekharaiah

**Affiliations:** 1 Jain University, Center for Post Graduate Studies, Department of Biotechnology, Bangalore, India

**Keywords:** L-Carnitine, Plasma, Antioxidant enzymes, Lipid peroxidation, Protein oxidation

## Abstract

**Objective:**

Plasma acts as a good indicator of oxidative stress in blood. L-Carnitine is an antioxidant that reduces metabolic stress in cells, thereby providing a protective effect against oxidative stress (OS). L-Carnitine as an additive in storage has not been explored. Thus, this study attempts to analyze the role of L-carnitine in blood storage solution, citrate phosphate dextrose adenine (CPDA)-1, through OS markers including antioxidant enzymes, lipid peroxidation, and protein oxidation.

**Materials and Methods:**

Blood was collected from male Wistar rats and stored in CPDA-1 solution with L-carnitine (10 mM, 30 mM, and 60 mM: groups LC 10, LC 30, and LC 60, respectively) and without L-carnitine (control group). Plasma was isolated every 5th day and the OS markers were analyzed.

**Results:**

Superoxide dismutase (SOD) and sulfhydryl (SH) increased over storage in controls, LC 30, and LC 60. Catalase increased in LC 30 and LC 60 during storage. Thiobarbituric acid reactive substances (TBARS) and protein carbonyl (PrC) levels in all groups increased initially and reduced towards the end of storage. SOD and SH levels were maintained while TBARS and PrC levels increased in LC 10.

**Conclusion:**

L-Carnitine was beneficial in terms of increased antioxidant capacity and SH and decreased lipid peroxidation. This forms the basis for further studies on L-carnitine as a constituent in storage solutions.

## Abstract (tr)

**Amaç:**

Plazma kanda oksidatif stresin iyi bir göstergesi olarak görev yapar. L-karnitin hücrelerde metabolik stresi azaltan böylece oksidatif strese (OS) karşı koruyucu etki sağlayan bir antioksidandır. L-Karnitinin depolamada katkı maddesi olarak kullanımı araştırılmamıştır. Bu nedenle, bu çalışmada kan depolama solüsyonu olan sitrat fosfat dekstroz adenin (CPDA)-1 ‘e eklenen L-karnitinin rolü antioksidan enzimler, lipid peroksidasyonu ve protein oksidasyonunu içeren OS göstergeleri aracılığı ile analiz edilmektedir.

**Gereç ve Yöntemler:**

Wistar sıçanlarından kan örneği alındı ve CPDA-1 solüsyonunda L-karnitin ile (10 mM, 30 mM, ve 60 mM: Sırasıyla LC 10, LC 30 ve LC 60 grupları) ve L-karnitinsiz (kontrol grup) olarak depolandı. Plazma her 5. günde izole edildi ve OS göstergeleri analiz edildi.

**Bulgular:**

Süperoksit dismutaz (SOD) ve sülfidril (SH) kontroller de (LC30 ve LC60) depolama boyunca arttı. Katalaz LC30 ve LC60 da depolama sırasında arttı. Tiyobarbitürik asit reaktif maddesi (TBARS) ve protein karbonil (PrC) düzeyleri tüm gruplarda başlangıçta arttı ve depolama sonuna doğru azaldı. SOD ve SH düzeyleri LC 10’da korunurken TBARS ve PrC düzeyleri arttı.

**Sonuç:**

L-Karnitin antioksidan kapasite ve SH artışı ile lipid peroksidasyonu azalması açısından faydalı idi. Bu durum, L-karnitinin depolama solüsyonlarında bileşen olarak ileri çalışmaları yapılması için bir temel oluşturmaktadır.

## INTRODUCTION

L-Carnitine (L-3 hydroxy-4-N-N-N-trimethylaminobutyrate) is an essential nutrient that the body uses to convert fat into energy. It is required for the transport of fatty acids from the cytosol into the mitochondria during breakdown of lipids via β-oxidation. It acts as an antioxidant that reduces metabolic stress in cells, thereby providing a protective effect against lipid peroxidation and oxidative stress (OS) in the phospholipid membrane and the myocardial and endothelial cells [1].

Disturbances in the redox state can cause OS, which is an imbalance between the production of reactive oxygen species (ROS) and the biological system’s natural ability to detoxify these intermediates or repair the resulting damage caused by them [2]. OS is also induced during storage of blood. The constituents of plasma change during storage due to cell metabolism and the activation of proteolytic activity [3]. These changes can be attributed to the depletion of glucose levels in plasma, cationic pump failure, hemoconcentration, and leakage of cell constituents and metabolites in erythrocytes [4].

Plasma reflects the overall OS environment in whole blood as it holds all the cellular components in suspension. Thus, plasma serves as a good candidate for assessing the changes occurring during whole blood storage. The study of d’Almeida et al. [5] reported that 1 week of rat blood storage is equivalent to 4 weeks of human blood storage. Thus, rat blood was used for studying storage lesions.

The ability of L-carnitine to combat OS was studied by Li et al. [1], where it was found that L-carnitine could protect hepatocytes through its antioxidant effect. L-Carnitine could reduce free radical-induced oxidative damage of intermittent hypoxia exposure, thus delaying muscle fatigue [6,7].

Various studies have reported on the effects of L-carnitine on other blood components (erythrocytes and platelets) [8,9,10,11]. Although there are reports on plasma storage [12,13,14,15], very few have focused on plasma isolated from stored blood. However, the influence of Carnitine on stored blood is still unclear. Thus, this study attempts to analyze the role of L-carnitine as a constituent in blood storage solution through OS markers (antioxidant enzymes, lipid peroxidation, and protein oxidation) in plasma.

## MATERIALS AND METHODS

Animal care and maintenance was in accordance with the Ethical Committee regulations (841/b/04/CPCSEA).

### Chemicals

Epinephrine, thiobarbituric acid (TBA), and bovine serum albumin (BSA) were purchased from Sigma-Aldrich Chemicals (St. Louis, MO, USA). All other chemicals used were of reagent grade and organic solvents were of spectral grade.

### Blood Sampling

Animals were lightly anesthetized with ether and restrained in dorsal recumbency as described earlier [16]. In brief, the syringe needle was inserted just below the xiphoid cartilage and slightly to the left of the midline. Blood was carefully aspirated from the heart into polypropylene collection tubes with citrate phosphate dextrose adenine (CPDA)-1.

### Experimental Design

Blood was drawn from 4-month-old male Wistar rats and stored over a period of 20 days at 4 °C in CPDA-1. The samples from 20 animals were divided into 4 groups of 5 animals each: i) controls, ii) LC 10 (samples with L-carnitine at a concentration of 10 mM), iii) LC 30 (samples with L-carnitine at a concentration of 30 mM), and iv) LC 60 (samples with L-carnitine at a concentration of 60 mM). Plasma was isolated from whole blood at regular intervals of 5 days and biomarkers, i.e. antioxidant enzymes, lipid peroxidation, and protein oxidation products, were assessed.

### Plasma Separation

Plasma was isolated in microcentrifuge tubes by centrifuging for 20 min at 1000 × g. The plasma was removed and stored in isotonic phosphate buffer at -20 °C for further assays [17].

### Antioxidant Enzymes

**Superoxide Dismutase [EC 1.15.1.1]:** Superoxide dismutase (SOD) was measured by the method of Mishra and Fridovich [18]. Plasma was added to carbonate buffer (0.05 M). Epinephrine was added to the mixture and absorbance was measured at 480 nm. SOD activity was expressed as the amount of enzyme that inhibited oxidation of epinephrine by 50%.

**Catalase [EC 1.11.1.6]:** Catalase (CAT) was determined by the method of Aebi [19]. Briefly, plasma with absolute alcohol was incubated at 0 °C. An aliquot was taken up with 6.6 mM H2O2 and the decrease in absorbance was measured at 240 nm. An extinction coefficient of 43.6 M cm-1 was used to determine enzyme activity.

### Lipid Peroxidation: Thiobarbituric Acid Reactive Substances

Thiobarbituric acid reactive substances (TBARS) content was determined by the method of Bar-Or et al. [20]. Plasma with 0.9% sodium chloride was incubated at 37 °C for 20 min, and then 0.8 M HCl containing 12.5% trichloroacetic acid (TCA) and 1% TBA was added and samples were kept in a boiling water bath for 20 min and cooled at 4 °C. Centrifugation was carried out at 1500 × g and absorbance was measured at 532 nm. TBARS content was calculated by using the extinction coefficient of 1.56x10^5^ M^-1^ cm^-1^.

### Protein Oxidation

**Protein carbonyls:** Protein carbonyl (PrC) content was determined by the method of Reznick and Packer [21]. PrC content was measured by forming a labeled protein hydrazone derivative using 2,4-dinitrophenyl hydrazine (DNPH), which was then quantified spectrophotometrically. Briefly, after precipitation of protein with an equal volume of 1% TCA, the pellet was resuspended in 10 mM DNPH. Samples were kept in the dark for 1 h. An equal volume of 20% TCA was added and left on ice for 10 min and then centrifuged at 3000 × g, and the pellet was washed with an ethanol-ethyl acetate mixture (1:1) to remove the free DNPH and lipid contaminants. The final pellet was dissolved in 6 M guanidine HCl in 133 mM Tris and absorbance was measured at 370 nm. PrC content was calculated by using the extinction coefficient of 20,000 M^-1^ cm^-1^.

### Protein Sulfhydryls

The protein sulfhydryl (P-SH) concentration in the proteins was measured as described by Habeeb [22]. In brief, 0.08 mol/L sodium phosphate buffer containing 0.5 mg/mL Na_2_-ethylenediaminetetraacetic acid and 2% SDS was added to the sample in each assay tube, and then 0.1 mL of 5,5’-DTNB was added and the solution was vortexed. Color was allowed to develop at room temperature and absorbance was measured at 412 nm. P-SH was calculated from the net absorbance and molar absorptivity, 13,600 M^-1^ L^-1^ cm^-1^.

### Protein

Protein was determined in the plasma by the method of Lowry et al. [23] using BSA as the standard.

### Statistical Analysis

Results are represented as mean ± standard error. The Kolmogorov-Smirnov test was performed for suitability of the data. Values between the groups (storage period) and subgroups (antioxidants) were analyzed by two-way ANOVA and differences were considered significant at p<0.05. The Bonferroni post-test was performed using GraphPad Prism 6 software.

## RESULTS

### Superoxide Dismutase

**Figure 1:**
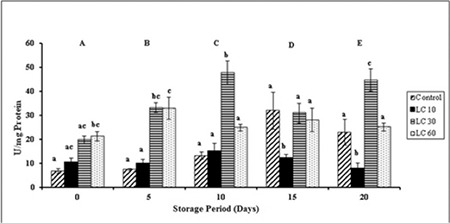
Superoxide dismutase activity in plasma isolated from stored blood. LC 10: L-carnitine 10 mM, LC 30: L-carnitine 30 mM, LC 60: L-carnitine 60 mM. Values are mean ± SE of five animals/group. Two-way ANOVA was performed between the groups and subgroups to analyze superoxide dismutase activity followed by the Bonferroni post-test, using GraphPad Prism 6 software. Changes between the groups are represented in upper case. Changes within the groups are represented in lower case. Those not sharing the same letters are significantly different.

Significant changes in SOD were observed in all groups with storage. SOD in controls increased during storage. SOD was maintained in LC 10 throughout the storage period. LC 30 and LC 60 samples showed increments in SOD over storage (Figure 1).

### Catalase

**Figure 2:**
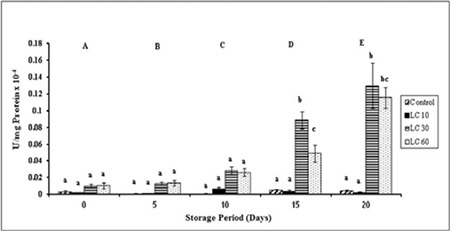
Catalase activity in plasma isolated from stored blood. LC 10: L-carnitine 10 mM, LC 30: L-carnitine 30 mM, LC 60: L-carnitine 60 mM. Values are mean ± SE of five animals/group. Two-way ANOVA was performed between the groups and subgroups to analyze catalase activity followed by the Bonferroni post-test, using GraphPad Prism 6 software. Changes between the groups are represented in upper case. Changes within the groups are represented in lower case. Those not sharing the same letters are significantly different.

Changes in CAT were significant with storage in all groups. Control and LC 10 CAT levels were maintained over storage. LC 30 and LC 60 levels were significantly increased at days 15 and 20 (Figure 2).

### Lipid Peroxidation - Thiobarbituric Acid Reactive Substances

**Figure 3:**
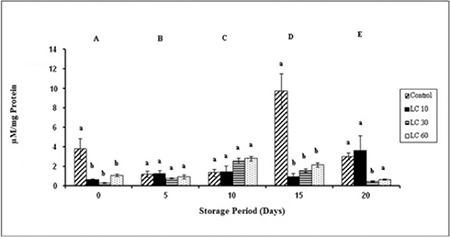
Thiobarbituric acid reactive substances in plasma isolated from stored blood. LC 10: L-carnitine 10 mM, LC 30: L-carnitine 30 mM, LC 60: L-carnitine 60 mM. Values are mean ± SE of five animals/group. Two-way ANOVA was performed between the groups and subgroups to analyze thiobarbituric acid reactive substances followed by the Bonferroni post-test, using GraphPad Prism 6 software. Changes between the groups are represented in upper case. Changes within the groups are represented in lower case. Those not sharing the same letters are significantly different.

Changes in TBARS were significant in controls during storage. TBARS peaked on day 15 in controls. An increase in TBARS was observed in LC 10 over storage. TBARS content was maximum on day 10 in LC 30 and LC 60 samples (Figure 3).

### Protein Oxidation

**Figure 4:**
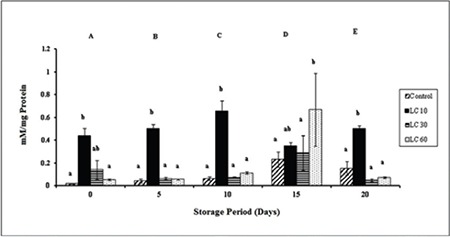
Protein carbonyls in plasma isolated from stored blood. LC 10: L-carnitine 10 mM, LC 30: L-carnitine 30 mM, LC 60: L-carnitine 60 mM. Values are mean ± SE of five animals/group. Two-way ANOVA was performed between the groups and subgroups to analyze protein carbonyl followed by Bonferroni post-test, using GraphPad Prism 6 software. Changes between the groups are represented in upper case. Changes within the groups are represented in lower case. Those not sharing the same letters are significantly different.

**Protein carbonyls:** Changes in PrC were significant in all groups with storage. PrC levels were increased at days 15 and 20 in controls. PrC was significantly higher than in the other groups throughout the storage period in LC 10. PrC peaked on day 15 in LC 30 and LC 60 samples (Figure 4).

**Figure 5:**
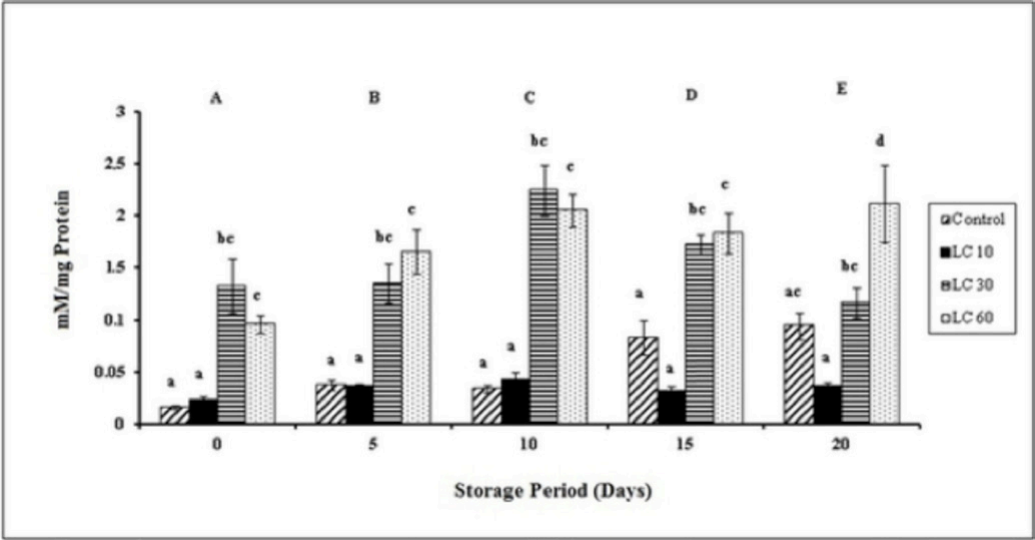
Protein sulfhydryls in plasma isolated from stored blood. LC 10: L-carnitine 10 mM, LC 30: L-carnitine 30 mM, LC 60: L-carnitine 60 mM. Values are mean ± SE of five animals/group. Two-way ANOVA was performed between the groups and subgroups to analyze protein sulfhydryl followed by Bonferroni post-test, using GraphPad Prism 6 software. Changes between the groups are represented in upper case. Changes within the groups are represented in lower case. Those not sharing the same letters are significantly different.

**Protein sulfhydryls:** P-SH levels varied significantly over storage. P-SH increased gradually throughout the storage period in controls. P-SH was maintained in LC 10 samples over the storage period. P-SH was significantly higher in LC 30 and LC 60 than controls and LC 10 (Figure 5).

## DISCUSSION

This study assessed whole blood through plasma, as it holds all the blood components in suspension. It gives an overall view of the OS microenvironment during storage.

SODs are a group of enzymes that catalyze the conversion of superoxide into H_2_O_2_ and O_2_. Increase in the activity of SOD is generally a sign of increased formation of superoxide radicals and thus elevated OS [24]. This was evident in our results of increased SOD activity during storage. L-Carnitine is a scavenger of free radicals and protects the cells from OS [25]. Similar results were observed in our study, where L-carnitine upregulated SOD [1,26]. SOD increased on day 10 and peaked on day 15 in controls, due to maximum ROS [27]. The decrease on day 20 in controls can be attributed to ROS overwhelming the antioxidant enzyme capacity and thus inactivating the enzyme. A similar trend was observed in LC 10 from day 15. SOD varied from day 10 onwards in LC 30, which can be attributed to the modulation of the enzyme activity by L-carnitine in proportion to superoxides.

CAT degrades H_2_O_2_ to H_2_O and O_2_. H_2_O_2_ can also be scavenged by glutathione peroxidase (GPX) [28]. CAT activity was low initially and increased over storage. This may be due to the activity of GPX scavenging H_2_O_2_ at lower concentrations, while CAT decomposes H_2_O_2_ only at high concentrations [29,30]. CAT levels were highest on day 15 in controls, similar to SOD, due to maximum ROS being produced on that day [23]. CAT expression in LC 10 was in accordance with the SOD levels, where the levels dropped on day 15. Li et al. [1] and Cao et al. [26] showed that L-carnitine increased CAT expression, which was also observed in our results. The increase in CAT in LC 30 and LC 60 can be attributed to L-carnitine’s ability to upregulate antioxidant enzyme activity.

TBARS, a biomarker of lipid peroxidation, is a reasonable reflection of a nonlipophilic peroxidation product, malondialdehyde [26]. The peak on day 15 in controls may be attributed to greater amounts of ROS generated [27]. L-Carnitine at concentrations of 30 mM and 60 mM reduced TBARS over storage. This may be due to L-carnitine’s ability to scavenge ROS and upregulate antioxidant enzymes at higher concentrations. L-Carnitine also has the property of preventing the accumulation of lipid peroxidation end products, hence causing the decrease in TBARS [31]. This was evident in our results of lipid peroxidation.

Oxidative cleavage of the protein backbone, oxidation of amino acids, or binding of aldehydes produced from lipid peroxidation produces PrC. It is formed early and circulates in the blood for longer periods as it is more stable than lipid peroxidation products [32]. Protein oxidation products are effective biomarkers of OS due to their long half-lives [33]. PrC increased in controls over storage, indicative of oxidative insult and protein damage. Addition of L-carnitine at 30 mM and 60 mM did not alter the PrC levels significantly, suggesting that L-carnitine could not alter the formation of carbonyls.

P-SH gets oxidized to disulfides, which is a reversible reaction. It is mainly present in the cysteine components of proteins and generally at lower concentrations in glutathione [34]. P-SH increased over storage in controls, which indicates that the endogenous antioxidant system could combat OS during storage. The increase in P-SH with L-carnitine indicates that it could protect sulfhydryl groups against oxidation or was effective in catalyzing the reversible change of disulfides to sulfhydryls [26].

Arduini et al. [11] reported L-carnitine to be beneficial at 5 mM in terms of increased ATP concentrations and reduced hemolysis over storage. However, our study showed that L-carnitine at 10 mM could not prevent protein oxidation and lipid peroxidation, but LC 30 and LC 60 had reduced oxidative damage through reduced TBARS and elevated P-SH and antioxidant enzymes (SOD and CAT).

## CONCLUSION

In conclusion, antioxidant enzymes in plasma could combat the ROS generated during storage. Our study showed that L-carnitine at higher concentrations can be further explored as a constituent of storage solutions as it significantly upregulated the antioxidant capacity of plasma and reduced oxidative damage during storage. Therefore, L-carnitine is a promising constituent in blood storage solutions.
